# Tumor necrosis is associated with increased alpha_v_beta_3 _integrin expression and poor prognosis in nodular cutaneous melanomas

**DOI:** 10.1186/1471-2407-8-362

**Published:** 2008-12-05

**Authors:** Ingeborg M Bachmann, Rita G Ladstein, Oddbjørn Straume, George N Naumov, Lars A Akslen

**Affiliations:** 1 The Gade Institute, Section for Pathology, University of Bergen, Haukeland University Hospital Bergen, Norway; 2 Department of Dermatology, Haukeland University Hospital, Bergen, Norway; 3 Department of Surgery, Children's Hospital and Vascular Biology Program, Harvard Medical School, Boston, MA, USA

## Abstract

**Background:**

Tumor necrosis and apoptotic activity are considered important in cancer progression, but these features have not been much studied in melanomas. Our hypothesis was that rapid growth in cutaneous melanomas of the vertical growth phase might lead to tissue hypoxia, alterations in apoptotic activity and tumor necrosis. We proposed that these tumor characteristics might be associated with changes in expression of cell adhesion proteins leading to increased invasive capacity and reduced patient survival.

**Methods:**

A well characterized series of nodular melanoma (originally 202 cases) and other benign and malignant melanocytic tumors (109 cases) were examined for the presence of necrosis, apoptotic activity (TUNEL assay), immunohistochemical expression of hypoxia markers (HIF-1 α, CAIX, TNF-α, Apaf-1) and cell adhesion proteins (α_v_β_3 _integrin, CD44/HCAM and osteopontin). We hypothesized that tumor hypoxia and necrosis might be associated with increased invasiveness in melanoma through alterations of tumor cell adhesion proteins.

**Results:**

Necrosis was present in 29% of nodular melanomas and was associated with increased tumor thickness, tumor ulceration, vascular invasion, higher tumor proliferation and apoptotic index, increased expression of α_v_β_3 _integrin and poor patient outcome by multivariate analysis. Tumor cell apoptosis did also correlate with reduced patient survival. Expression of TNF-α and Apaf-1 was significantly associated with tumor thickness, and osteopontin expression correlated with increased tumor cell proliferation (Ki-67).

**Conclusion:**

Tumor necrosis and apoptotic activity are important features of melanoma progression and prognosis, at least partly through alterations in cell adhesion molecules such as increased α_v_β_3 _integrin expression, revealing potentially important targets for new therapeutic approaches to be further explored.

## Background

Multiple features are important for the progress of cutaneous melanoma, like alterations of tumor cell proliferation and cell cycle regulation, cell adhesion proteins, and tumor associated angiogenesis [1-3]. Although necrosis and tumor cell apoptosis are strongly related to the behaviour of malignant tumors, these characteristics have not been well studied in human melanomas. We therefore examined whether these features are associated with the development and clinical progression of melanocytic tumors. Selected markers related to tissue hypoxia were examined, *e.g*. HIF-1α, CAIX, TNF- α, and Apaf-1. We also asked whether necrosis was associated with alterations of tumor phenotype, and the expression of cell adhesion markers (α_v_β_3 _integrin, CD44/HCAM, osteopontin) was determined.

Whereas HIF-1α expression has been associated with decreased survival in cancers of the breast and other sites [4-7], little is known about its role in melanoma. Expression of CAIX, another marker of tissue hypoxia [8], has been associated with tumor progression in some cancers [9,10], but melanomas were negative in one small study [11]. TNF-α is an important mediator of apoptosis via the "extrinsic pathway" [12], but there are limited data on its role in human melanoma. Apaf-1 is involved in the mitochondrial apoptotic pathway, and studies have reported reduced expression in advanced melanoma and a possible relationship with melanoma chemoresistance [13-15].

Adhesion molecules are involved in the regulation of cellular migration and tumor invasion [16]. We here asked whether necrosis and tissue hypoxia are associated with alterations in cell adhesion markers. Among these, the integrin receptors are important for the binding of cells to extracellular matrix components. Experimentally induced HIF leads to upregulation of α_v_β_3 _integrin followed by increased invasive capacity [17], supporting our hypothesis. In melanomas, the α_v_-integrin subunit is widely expressed, whereas β_3 _is mainly restricted to vertical growth phase tumors [16] and associated with poor prognosis [18,19]. CD44/HCAM is also a cell adhesion protein interacting with matrix components [20], and expression in melanoma cells has been linked to poor prognosis in some [21,22] but not all studies [23-25]. The secreted phosphoprotein osteopontin, which binds to both α_v_β_3 _integrin and CD44 receptors, might influence tumor cell attachment and migration [26]. In mice, osteopontin deficiency suppresses growth of melanoma cells implanted in the bone marrow [27]. In metastatic melanoma, Zhou et al. found by cDNA microarray analysis that osteopontin was the most overexpressed gene when compared with benign nevi [28]. Recently, osteopontin expression was shown to be a marker of lymph node metastases and poor patient prognosis in melanoma [29].

In our present study, we found that both hypoxia and cell adhesion markers showed an increased expression from benign nevi to melanomas, indicating an importance of these pathways in early melanoma development. In established melanoma, tumor necrosis was related to increased expression of α_v_-integrin, and both were strongly associated with features of aggressive tumors and poor patient outcome as shown by multivariate survival analysis.

## Methods

### Patient samples

This population-based series has been described in detail elsewhere [1]. Briefly, 202 cutaneous melanomas of the nodular type occurring during 1981–97 were initially included. For the present study, 133 cases had sufficient material left in the tissue microarray blocks to be studied. The presence of a vertical growth phase and the lack of a radial growth phase, *i.e*. adjacent in situ or microinvasive component, were used as inclusion criteria for this series. In addition, 58 paired metastases (local, regional lymph nodes, distant) were examined. Clinico-pathologic characteristics and some survival data have previously been reported [1]. For comparison, 31 cases of consecutive benign melanocytic nevi and 20 cases of superficial spreading melanomas (median thickness, 1.7 mm) were included to examine these markers in different stages of melanocytic tumor progression. The Norwegian Data Inspectorate and the Regional Committee for Ethics in Research (Health Region III) have approved this study.

For the present study, HE-slides were re-examined and the presence of necrotic areas was recorded (see later). Cases with sufficient material left in the TMA blocks (range 129–133; differences due to drop-outs) were examined for the expression of hypoxia related markers (HIF-1α, CAIX, TNF-α and Apaf-1) and selected cell adhesion markers (α_v_-integrin, β_3_-integrin, CD44/HCAM, osteopontin). Apoptotic activity was estimated by the TUNEL assay.

### Clinico-pathologic variables

The following variables were recorded: date of histologic diagnosis, sex, age at diagnosis, anatomical site of the primary tumor, and presence of metastases at diagnosis. The following histological features were also included: tumor thickness according to Breslow [30], level of invasion according to Clark [31], microscopic ulceration and vascular invasion [32].

### Tumor necrosis

The original HE-slides were re-examined (I.M.B., L.A.A.) and presence of tumor necrosis was evaluated. Necrosis was recorded as present when an area of at least 1/4 high power field (0.07 mm^2^) was occupied by necrotic cells and sparse when clusters of at least five necrotic cells (but less than 1/4 HPF) were observed. Cases presenting scattered necrotic cells of less than five in a cluster were regarded as negative.

### Tissue Microarray (TMA)

The technique of TMA is well established [33] and validated [1,34,35]. For TMA construction [33,35], representative tumor areas were identified on HE-stained slides, generally located at the suprabasal areas of the primary tumors [34]. Three parallel tissue cylinders (diameter 0.6 mm) were sampled from each case. Sections of the resulting TMA blocks (5 μm) were then made by standard technique.

### Apoptotic index (TUNEL assay)

Fluorescent terminal deoxyuridine triphosphate (dUTP) nick-end labeling (TUNEL) staining was performed on the TMA sections using the In situ Cell Death Detection Kit (Roche Diagnostic GmbH, Mannheim, Germany) according to the manufacturer's instructions. Briefly, the sections were deparaffinized in xylene and rehydrated in ethanol before enzymatic retrieval with Proteinase K (Dako Cytomation, Copenhagen, Denmark), followed by blocking with 4% bovine serum albumin and incubation with a mixture of TdT solution (enzyme solution) and labelled dUTP solution (label solution) in 37°C for 1 hour in a dark humified chamber. After incubation in alkaline phosphatase converter, fast blue was used as substrate and hematoxylin eosin as counterstain. The number of TUNEL-positive tumor cell nuclei was counted in 200 cells by light microscopy and use of an ocular grid, and an apoptotic index (AI) was determined as the percentage of tumor cells being positive. In subsequent statistical analyses, AI was divided by the upper quartile (cut-off: AI ≤ 3.5 % versus > 3.5 %). A subset of the cases were blindly scored by two of the authors, showing good correlation (Pearson's correlation coefficient 0.91, p < 0.0001).

### Immunohistochemistry

The immunohistochemical staining was performed on thin sections (5 μm) of paraffin-embedded archival tissue, using TMA sections as described. Samples were dewaxed and rehydrated with xylene/ethanol before microwave antigen retrieval and antibody incubation, using protocols optimized for each antibody (Table 1). The staining procedures, except the protocol for HIF-1α, were all performed using the EnVision labelled polymer method, with commercial available kits (DakoCytomation, Copenhagen, Denmark).

**Table 1:** Immunohistochemical staining methods

**Antibody**	**Provider**	**Epitope retrieval**	**Dilution**	**Incubation**	**Signal amplification**
HIF-1α, MS-1164, clone 67 (MoAb)	Neomarkers	15 min in TrisEDTA buffer (pH = 9) at 350 W	1:100	60 min RT^a^	CSA (DAKO)
CAIX, NB 100–417 (pAb)	Novus Biologicals	15 min in citrate buffer (pH = 6) at 350 W	1:100	60 min RT	no
TNF-α, ab 6671 (pAb)	Abcam	15 min in TrisEDTA buffer (pH = 9) at 350 W	1:200	Overnight 4°C	no
Apaf-1, RB 9236 (pAb)	Neomarkers	15 min in citrate buffer (pH = 6) at 350 W	1:100	60 min RT	no
CD51/αv-integrin, MS 1774, clone CJ00 (MoAb)	Neomarkers	15 min in EDTA buffer (pH = 8) at 350 W	1:20	60 min RT	no
CD61/β3-integrin, MS 1842, clone Y2/51 (MoAb)	Neomarkers	15 min in citrate buffer (pH = 6) at 350 W	1:25	Overnight 4°C	no
CD44/HCAM, MS 668, clone 156-3C11 (MoAb)	Neomarkers	15 min in citrate buffer (pH = 6) at 350 W	1:200	60 min RT	no
Osteopontin (MoAb)	Gift from prof. Ann F. Chambers	15 min in citrate buffer (pH = 6) at 350 W	1:500	60 min RT	No

^a^RT = room temperatur

Information on Ki-67, p16, p53 [1], tumor associated angiogenesis estimated by microvessel density (MVD) [36], E-cadherin, N-cadherin, P-cadherin, and β-catenin [2] from our previous studies was included for comparison.

### Evaluation of staining

**Figure 1:**
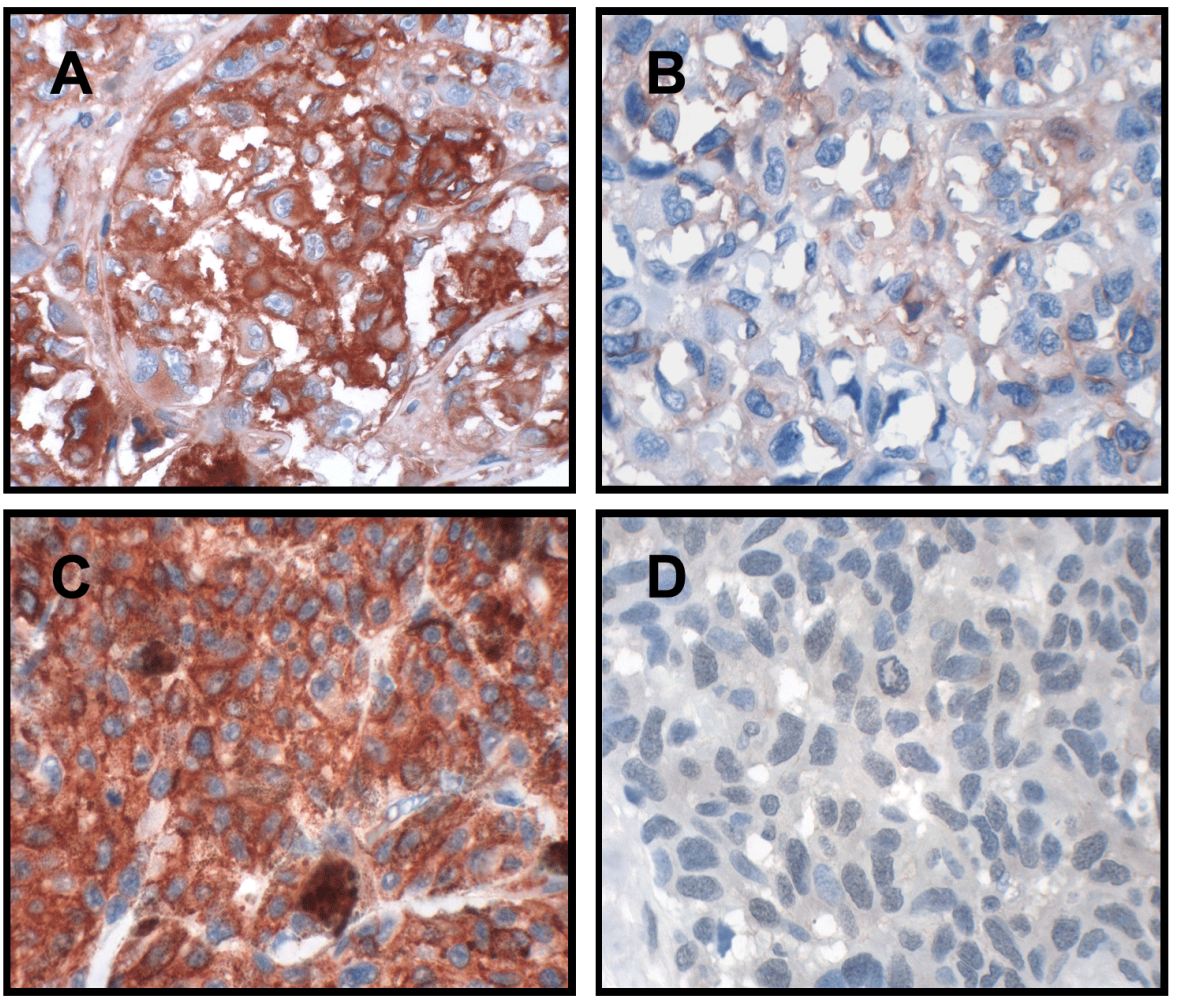
Immunohistochemical staining showing (A) strong and (B) weak expression of αv-integrin, and (C) strong and (D) weak expression of β_3 _integrin in primary nodular melanoma.

The staining of HIF-1α was restricted to the nuclei of tumor cells, as described by others [37]. The staining for CAIX, TNF-α and Apaf-1 was mainly cytoplasmic and homogenously distributed. CD44 showed a membranous staining pattern. The staining of α_v _and β_3 _integrin (Fig. 1), as well as the osteopontin protein, was most pronounced in the membrane and cytoplasm of the tumor cells.

The immunohistochemical staining was recorded using a semi-quantitative and subjective grading, considering both the intensity of staining and the proportion of tumor cells showing unequivocal positive reaction. Staining intensity: 0, no staining; 1, weak staining; 2, moderate staining; and 3, strong staining. Staining area: 0, no staining; 1, positive staining in < 10% of tumor cells; 2, positive staining in 10–50% of tumor cells; 3, positive staining in > 50% of tumor cells. A staining index (SI) was then calculated as previously reported, as staining intensity times positive area [38]. In subsequent statistical analyses, cut-off points for SI categories were mainly based on median or quartile values, considering also the frequency distribution curve and size of subgroups for each marker, as well as number of cases and events (follow-up information) when determining the final cut-off points between low and high expression (two categories). In survival analysis, subgroups (based on quartiles) with similar survival were subsequently merged. HIF-1α was categorized as negative (SI 0–1) and positive (SI ≥ 2), whereas expression of CAIX, Apaf-1, α_v_-integrin, and osteopontin was considered low (SI ≤ 4) and high (SI > 4). TNF-α and β_3_-integrin were divided by SI < 3 (low) and ≥ 3 (high), and CD44 was divided by ≤ 6 (low) and > 6 (high).

### Follow-up

Complete information on patient survival and time and cause of death was available in all 202 cases [2]. Last date of follow-up was December 31, 1999, and median follow-up time for survivors was 89 months (range 24–221). Clinical follow-up (with respect to recurrences) was not carried out in 14 patients (predominantly older age), and 21 patients were not treated with complete local excision. Thus, recurrence-free survival was available in 167 patients. During the follow-up period, 72 patients (36%) died of malignant melanoma, and 45 (22%) died of other causes. Of the 167 radically treated patients with data on recurrence-free survival, 74 (44%) had recurrent disease.

### Statistics

Analyses were performed using the SPSS statistical package, version 12.0 (SPSS Inc, Chicago, IL). Associations between different categorical variables were assessed by Pearson's chi-square test. Continuous variables not following the normal distribution were compared between two groups using the Mann-Whitney U test. The Wilcoxon signed rank test was used to compare related samples. Univariate analyses of time to death due to malignant melanoma or time to recurrence (recurrence-free survival) were performed using the product-limit procedure (Kaplan-Meier method), and differences between categories were estimated by the log-rank test, with date of histological diagnosis as the starting point. Patients who died of other causes were censored at the date of death. The influence of co-variates on patient survival was analysed by the proportional hazards method, and tested by the likelihood ratio (lratio) test. The level for inclusion in Cox' multivariate regression analyses was P = 0.10, and the co-variates included (step one) were tumor thickness according to Breslow, Clark's level of invasion, tumor ulceration, vascular invasion, p16 expression and tumor cell proliferation assessed by Ki-67, in addition to tumor necrosis and markers of cell adhesion and hypoxia described here.

## Results

### Tumor necrosis

Necrosis was not present in benign nevi. In nodular melanomas, tumor necrosis (sparse or significant) was observed in 57 (29%) of the cases and was associated with increased tumor thickness (median thickness 5.2 mm when necrosis was present, as compared to 3.0 mm in cases without necrosis; p < 0.0001), and with increased tumor cell proliferation by Ki-67 (35% versus 25% positive nuclei; p = 0.007). Necrosis was also associated with tumor ulceration (p < 0.0001) and vascular invasion (p = 0.003), with 68 % of necrotic tumors being ulcerated compared to 32% of tumors without necrosis. For vessel invasion, the corresponding figures were 33% versus 15%.

Tumors with necrotic areas had a slightly higher angiogenesis as estimated by microvessel density, 22.0 microvessels per mm^2 ^(median) in tumors with necrosis, compared with 19.0 among the rest (p = 0.031). Also, tumors with necrosis were associated with alterations of cell adhesion molecules, as shown by increased expression of cytoplasmic P-cadherin and weaker nuclear β-catenin staining (p = 0.017 and p = 0.002, respectively), both being associated with aggressive melanomas in our previous study [2].

### Apoptotic index

Benign melanocytic nevi only rarely contained apoptotic cells (median apoptotic index, AI = 0). When comparing nevi with melanomas (superficial and nodular melanomas combined), there was markedly higher AI among the latter (p = 0.004) (Table 2), whereas there was no difference in AI when comparing primary nodular melanomas with their corresponding metastases. Median AI in nodular melanomas was 1.5 %. Tumors with necrosis had a higher apoptotic index (median 2.5) than tumors without necrosis (median 1.25; p = 0.040). Increased AI was also associated with tumor ulceration (p = 0.038), and localization on the trunk (p = 0.040). Loss of p16 protein expression was associated with high AI (p = 0.008). Further, high AI (> 3.5 %, upper quartile) was significantly associated with reduced angiogenesis by MVD (p = 0.05), and with alterations of cell adhesion markers, such as strong expression of the cell adhesion marker β_3_-integrin (p = 0.027).

**Table 2:** Expression of cell adhesion proteins in benign melanocytic nevi and cutaneous melanoma

**Cell adhesion markers**	No. of cases		p^a^
		
	Nevi	NM^b ^+ SSM^c^	
**α_v_-integrin**			
weak^d^	15	42	
strong	16	104	0.034
**β3-integrin**			
weak^d^	21	53	
strong	10	97	0.001
**CD44**			
weak^d^	8	69	
strong	22	77	0.038
**osteopontin**			
weak^d^	26	60	
strong	5	88	< 0.0001

^a^Chi-square test

^b^Nodular melanoma

^c^Superficial spreading melanoma

^d^Cut-point median (SI)

The relative impact of tumor cell proliferation versus apoptotic activity was estimated by the ratio of Ki-67 and AI (Proliferation ratio = Ki-67/AI). High proliferation ratio (above 7.4, cut-point lower quartile) was associated with increased tumor thickness (p = 0.011).

### Cell adhesion related markers

Expression of the α_v _and β_3 _integrin subunits and of osteopontin was stronger in melanomas than in benign nevi (p = 0.034, p = 0.001 and p < 0.0001, respectively), whereas the expression of CD44 was weaker in melanomas than in nevi (p = 0.038) (Table 2). In contrast, β_3 _integrin expression was significantly decreased in melanoma metastases in reference to the corresponding primary nodular melanoma tumors (p < 0.0001). Among nodular melanomas, the α_v _and β_3 _integrin subunits were significantly co-expressed (p = 0.003). Strong α_v _expression was associated with presence of tumor necrosis, (p = 0.010), increasing tumor thickness (p = 0.002), and high levels of cytoplasmic P-cadherin expression (p = 0.009). Strong expression of β_3 _integrin was associated with tumor necrosis (p = 0.001), increased tumor thickness (p < 0.0001), tumor ulceration (p = 0.003), vascular invasion (p = 0.005), and tumor cell proliferation by Ki-67 expression (p = 0.019).

CD44 was significantly co-expressed with α_v_-integrin (p = 0.011) in nodular melanomas. Osteopontin expression was associated with β_3_-integrin staining (p = 0.028), increased tumor cell proliferation by Ki-67 (p = 0.023), and was significantly increased in metastatic lesions compared with their corresponding primary nodular melanomas (p < 0.0001).

### Hypoxia related markers

Expression of selected hypoxia markers HIF-1α (p = 0.002), CAIX (p < 0.0001), TNF-α (p = 0.008) and Apaf-1 (p = 0.002) was significantly stronger in melanomas than in benign nevi (Table 2). Among primary nodular melanomas, increased tumor thickness was associated with stronger expression of hypoxia markers TNF-α (p = 0.028) and Apaf-1 (p = 0.05) (Table 3), whereas tumor ulceration was associated with stronger expression of hypoxia markers HIF-1α (p = 0.05) and CAIX (p = 0.03). Expression of Apaf-1, a marker of the mitochondrial apoptotic pathway, was strongly associated with increased expression of adhesion markers α_v_-integrin (p = 0.001) and CD44 (p = 0.006). Also, Apaf-1 was stronger in metastatic lesions when compared with primary nodular melanomas (p = 0.002).

**Table 3:** Estimated 5- and 10-year survival rates for patients with vertical growth phase melanoma (product-limit method) by tumor necrosis, apoptotic index and α_v_-integrin, using death from melanoma as end-point

Variables	No. of cases	Estimated survival rates (%)		p^a^
			
		5 years	10 years	
**Tumor necrosis**				
absent	142	76	64	< 0.0001
present	57	45	35	
**Apoptotic index**				
low^b^	97	66	61	0.016
high	30	49	16	
**α_v_-integrin**				
weak^c^	39	80	80	0.006
strong	91	56	42	

^a^Log-rank test

^b^Cut-point upper quartile (SI)

^c^Cut-point median (SI)

### Survival analyses

**Figure 2:**
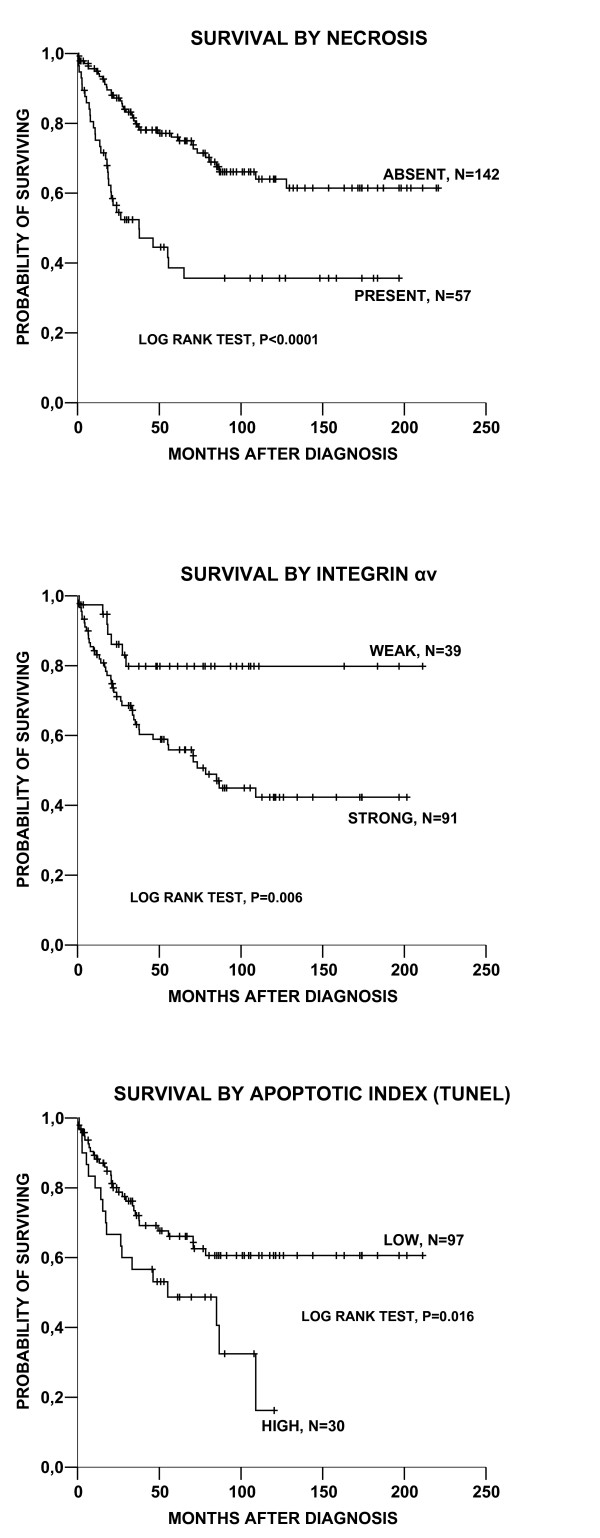
Survival curves according to the Kaplan-Meier method by (A) absence or presence of necrosis, (B) expression of integrin α_v_, and (C) apoptotic index (AI) in nodular melanoma with death of the disease as end-point.

Presence of necrosis was associated with significantly reduced overall survival (Table 3); (Fig. 2), as well as reduced recurrence-free survival (p = 0.016). High apoptotic activity (AI) was also significantly associated with reduced patient survival in univariate analysis (Table 3; Fig. 2), but not with reduced recurrence free survival (not shown).

Among the cell adhesion markers, high levels of α_v_-integrin expression was associated with significantly reduced survival (p = 0.006) (Table 3; Fig. 2). The same trend, although non-significant, was seen regarding expression of the β_3 _subunit (p = 0.13).

Among the hypoxia related markers, increased expression of HIF-1α (p = 0.061), TNF-α (0.059) and Apaf-1 (0.095) tended to be associated with reduced survival, although of borderline significance. Strong expression of HIF-1α was significantly associated with reduced recurrence-free survival, with 61% of the patients experiencing relapse within 5 years when HIF-1α was strong versus 50% of patients when HIF-1α was weak (p = 0.024).

In multivariate analysis, tumor necrosis turned out to be a strong and independent prognostic factor (HR 2.3, p = 0.001) when included along with basic prognostic factors like tumor thickness (using median as cut-off value, i.e ≤ vs > 3.55 mm), Clark's level of invasion, tumor ulceration, vascular invasion, p16 expression and tumor cell proliferation by Ki-67. When including these variables and necrosis along with markers of cell adhesion and hypoxia (all with p ≤ 0.10 in univariate analysis), both necrosis and α_v_-integrin expression remained as independent variables (final model shown in Table 4).

**Table 4:** Multivariate survival analysis (Cox' proportional hazards method) for patients with vertical growth phase melanoma, using tumor related death as end-point

Variables	Categories	n	HR^a^	95 % CI	p-value^b^
Clark's level of invasion	II, III, IV	101	1		
	V	26	2.5	1.3–4.7	0.007
Tumor necrosis	absent	86	1		
	present	41	2.7	1.5–4.8	0.001
αv-integrin expression	low^c^	37	1		
	high	90	2.3	1.0–5.1	0.049
p16 expression	high^c^	63	1		
	low	64	2.5	1.4–4.5	0.003

^a^Hazard ratio

^b^Lratio test

^c^Cut-point median SI

In this series of nodular melanomas, Clark's level of invasion has a somewhat stronger impact on survival than tumor thickness in multivariate analysis. When excluding Clark's level of invasion from the model, tumor thickness gains independent prognostic impact.

## Discussion

The importance of tumor necrosis and apoptotic activity for melanoma development and progression has not been well examined. In our study, necrosis was present in 29 % of nodular melanomas and was strongly associated with other features of aggressive tumors like ulceration, vascular invasion, and reduced patient survival as shown by multivariate analyses. Increased apoptotic rate was also associated with impaired prognosis. We here asked whether the presence of tumor necrosis, as a morphologic marker of tissue hypoxia [39], was associated with alterations in cell adhesion molecules and more aggressive melanoma features. As a novel finding, we found a strong association between necrosis and increased expression of the adhesion molecules α_v _and β_3 _integrins, suggesting a relationship between tissue hypoxia and more invasive melanomas. Experimental studies of melanoma cell lines have previously shown increased expression of α_v_β_3 _integrin after exposure to hypoxia [17]. Further, in studies of melanoma xenografts, up-regulation of hypoxia-related gene products has been associated with increased metastatic capacity [40]. In our study, we found that necrosis was associated with increased expression of both α_v _and β_3_integrin subunits, thus validating the findings from cell lines in human melanoma samples. It is hypothesized that tumor hypoxia induces or selects for more aggressive tumor features such as increased invasive properties and metastatic capacity. Integrin subunits such as α_v _and β_3 _might play an important role in this phenotypic shift. Both experimental [41] and clinical data [18,19,42] point at β_3 _integrin as a marker of melanoma growth and progression, whereas the role of α_v_-integrin has been less studied. Some reports indicate that α_v _promotes melanoma invasion [43], but others disagree [19,44]. Our present findings indicate a significant impact of α_v_-integrin on melanoma progress, as supported by associations with tumor thickness and presence of necrosis, and we also show for the first time that α_v _expression is associated with reduced patient survival by multivariate analysis. These findings might be of clinical importance, since α_v _blocking agents with anti-tumor and anti-angiogenic properties have been proposed as a treatment option for melanoma patients [45,46].

Presence of necrosis was associated with altered expression of α_v_and β_3 _integrins which are considered important for tumor cell invasion. We recently found that other cell adhesion markers appear to be significant for the invasion and prognosis of cutaneous melanoma, most notably a shift towards expression of P-cadherin and loss of nuclear β-catenin [2]. In the present study, both cytoplasmic P-cadherin staining and loss of nuclear β-catenin were significantly associated with tumor necrosis. Together these findings suggest even further that tumor necrosis may induce a shift in cell adhesion profile towards a more invasive phenotype. Alternatively, hypoxia might induce a selection of more aggressive tumor cell clones with different molecular signatures.

Expression of selected hypoxia markers (HIF-1α, CAIX, TNF-α, Apaf-1) was significantly stronger in melanomas than in benign nevi, indicating possible roles in early melanoma development. In our series of nodular melanomas, these markers also revealed some associations with features of aggressive tumors like increased tumor thickness and ulceration, and expression of two markers (TNF-α, Apaf-1) were further increased in melanoma metastases. However, cell adhesion markers (α_v _and β_3 _integrins) showed stronger associations with progression markers and patient outcome.

Our study indicates that increased apoptotic index correlates with melanoma development and poor patient outcome in nodular melanoma. In line with this observation, expression of Apaf-1, a marker of the mitochondrial apoptotic pathway, was increased from melanocytic nevi to primary melanomas and even more so in melanoma metastases. In contrast, previous studies have indicated loss of Apaf-1 in melanoma cells when compared to nevi, considered to represent an indication of reduced apoptotic capacity in invasive melanoma [13,14,47]. Apaf-1 expression was also associated with increased tumor thickness and strong staining of the pro-invasive α_v_-integrin. Taken together, our findings suggest that higher apoptotic activity is taking place in the more advanced melanomas, but the proliferative capacity is increasing even more, in favour of tumor growth and progression. This is underlined by our finding of an association between high proliferation ratio and increased tumor thickness, indicating relatively higher proliferation rates in tumors with abundance of apoptoses. Smaller studies of apoptosis in melanocytic tumors and basal cell carcinomas have indicated similar findings [48,49]. Thus, novel therapeutic approaches targeting cancer cells by inducing up-regulation of apoptosis [50] could be attractive in these tumors.

Recent gene array studies have identified osteopontin as highly over-expressed in metastatic melanoma when compared to benign nevi [28]. This is confirmed in our present study of protein expression, where osteopontin staining was increased from benign nevi to malignant melanomas. Expression was even stronger in melanoma metastases compared with corresponding primary tumors, in contrast to the study by Zhou et al., where no such correlation was reported [28]. The level of osteopontin expression was associated with both increased tumor cell proliferation and apoptotic rate, although not with patient outcome, contrasting another recent study of melanomas [29]. The mechanism of action is less clear, as there were no significant associations with its receptors α_v_β_3_integrin and CD44.

## Conclusion

Our findings indicate that hypoxia markers like tumor necrosis and increased apoptosis are associated with the progress of vertical growth phase melanoma of the nodular type. The prognostic significance of α_v_-integrin is reported for the first time, and underscores the importance of cell-matrix interactions in melanoma invasion and metastasis, supporting this as a potentially important therapy target that should be further investigated.

## Competing interests

The authors declare that they have no competing interests.

## Authors' contributions

LAA contributed to design of the study, histological review of the cases, interpretation of data, writing of the manuscript, and provided the funding. GNN participated in study design, coordination and writing of the manuscript. RGL participated in interpretation of data and writing of the manuscript. OS made the tissue arrays, collected follow-up data and patient demographics, and helped to design the study. IMB contributed to study design, laboratory work, interpretation of data, statistical analyses, and writing of the manuscript. All authors approved the final manuscript.

## Pre-publication history

The pre-publication history for this paper can be accessed here:



## References

[B1] Straume O, Sviland L, Akslen LA (2000). Loss of nuclear p16 protein expression correlates with increased tumor cell proliferation (Ki-67) and poor prognosis in patients with vertical growth phase melanoma. Clin Cancer Res.

[B2] Bachmann IM, Straume O, Puntervoll HE, Kalvenes MB, Akslen LA (2005). Importance of P-cadherin, beta-catenin, and Wnt5a/frizzled for progression of melanocytic tumors and prognosis in cutaneous melanoma. Clin Cancer Res.

[B3] Hsu M, Andl T, Li G, Meinkoth JL, Herlyn M (2000). Cadherin repertoire determines partner-specific gap junctional communication during melanoma progression. J Cell Sci.

[B4] Talks KL, Turley H, Gatter KC, Maxwell PH, Pugh CW, Ratcliffe PJ, Harris AL (2000). The expression and distribution of the hypoxia-inducible factors HIF-1alpha and HIF-2alpha in normal human tissues, cancers, and tumor-associated macrophages. Am J Pathol.

[B5] Dales JP, Garcia S, Meunier-Carpentier S, Andrac-Meyer L, Haddad O, Lavaut MN, Allasia C, Bonnier P, Charpin C (2005). Overexpression of hypoxia-inducible factor HIF-1alpha predicts early relapse in breast cancer: retrospective study in a series of 745 patients. Int J Cancer.

[B6] Nakanishi K, Hiroi S, Tominaga S, Aida S, Kasamatsu H, Matsuyama S, Matsuyama T, Kawai T (2005). Expression of hypoxia-inducible factor-1alpha protein predicts survival in patients with transitional cell carcinoma of the upper urinary tract. Clin Cancer Res.

[B7] Swinson DE, Jones JL, Cox G, Richardson D, Harris AL, O'Byrne KJ (2004). Hypoxia-inducible factor-1 alpha in non small cell lung cancer: relation to growth factor, protease and apoptosis pathways. Int J Cancer.

[B8] Potter C, Harris AL (2004). Hypoxia inducible carbonic anhydrase IX, marker of tumour hypoxia, survival pathway and therapy target. Cell Cycle.

[B9] Chia SK, Wykoff CC, Watson PH, Han C, Leek RD, Pastorek J, Gatter KC, Ratcliffe P, Harris AL (2001). Prognostic significance of a novel hypoxia-regulated marker, carbonic anhydrase IX, in invasive breast carcinoma. J Clin Oncol.

[B10] Giatromanolaki A, Koukourakis MI, Sivridis E, Pastorek J, Wykoff CC, Gatter KC, Harris AL (2001). Expression of hypoxia-inducible carbonic anhydrase-9 relates to angiogenic pathways and independently to poor outcome in non-small cell lung cancer. Cancer Res.

[B11] Ivanov S, Liao SY, Ivanova A, Danilkovitch-Miagkova A, Tarasova N, Weirich G, Merrill MJ, Proescholdt MA, Oldfield EH, Lee J, Zavada J, Waheed A, Sly W, Lerman MI, Stanbridge EJ (2001). Expression of hypoxia-inducible cell-surface transmembrane carbonic anhydrases in human cancer. Am J Pathol.

[B12] Reed JC (2000). Mechanisms of apoptosis. Am J Pathol.

[B13] Dai DL, Martinka M, Bush JA, Li G (2004). Reduced Apaf-1 expression in human cutaneous melanomas. Br J Cancer.

[B14] Niedojadlo K, Labedzka K, Lada E, Milewska A, Chwirot BW (2006). Apaf-1 expression in human cutaneous melanoma progression and in pigmented nevi. Pigment Cell Res.

[B15] Campioni M, Santini D, Tonini G, Murace R, Dragonetti E, Spugnini EP, Baldi A (2005). Role of Apaf-1, a key regulator of apoptosis, in melanoma progression and chemoresistance. Exp Dermatol.

[B16] McGary EC, Lev DC, Bar-Eli M (2002). Cellular adhesion pathways and metastatic potential of human melanoma. Cancer Biol Ther.

[B17] Cowden Dahl KD, Robertson SE, Weaver VM, Simon MC (2005). Hypoxia-inducible factor regulates alphavbeta3 integrin cell surface expression. Mol Biol Cell.

[B18] Hieken TJ, Farolan M, Ronan SG, Shilkaitis A, Wild L, Das Gupta TK (1996). Beta3 integrin expression in melanoma predicts subsequent metastasis. J Surg Res.

[B19] Trikha M, Timar J, Zacharek A, Nemeth JA, Cai Y, Dome B, Somlai B, Raso E, Ladanyi A, Honn KV (2002). Role for beta3 integrins in human melanoma growth and survival. Int J Cancer.

[B20] Lesley J, Hyman R, Kincade PW (1993). CD44 and its interaction with extracellular matrix. Adv Immunol.

[B21] Dietrich A, Tanczos E, Vanscheidt W, Schopf E, Simon JC (1997). High CD44 surface expression on primary tumours of malignant melanoma correlates with increased metastatic risk and reduced survival. Eur J Cancer.

[B22] Sviatoha V, Rundgren A, Tani E, Hansson J, Kleina R, Skoog L (2002). Expression of CD40, CD44, bcl-2 antigens and rate of cell proliferation on fine needle aspirates from metastatic melanoma. Cytopathology.

[B23] Schaider H, Soyer HP, Heider KH, Hofmann-Wellenhof R, Zatloukal K, Smolle J, Kerl H (1998). CD44 and variants in melanocytic skin neoplasms. J Cutan Pathol.

[B24] Karjalainen JM, Tammi RH, Tammi MI, Eskelinen MJ, Agren UM, Parkkinen JJ, Alhava EM, Kosma VM (2000). Reduced level of CD44 and hyaluronan associated with unfavorable prognosis in clinical stage I cutaneous melanoma. Am J Pathol.

[B25] Ichikawa T, Masumoto J, Kaneko M, Saida T, Sagara J, Taniguchi S (1998). Moesin and CD44 expression in cutaneous melanocytic tumours. Br J Dermatol.

[B26] Sodek J, Zhu B, Huynh MH, Brown TJ, Ringuette M (2002). Novel functions of the matricellular proteins osteopontin and osteonectin/SPARC. Connect Tissue Res.

[B27] Ohyama Y, Nemoto H, Rittling S, Tsuji K, Amagasa T, Denhardt DT, Nifuji A, Noda M (2004). Osteopontin-deficiency suppresses growth of B16 melanoma cells implanted in bone and osteoclastogenesis in co-cultures. J Bone Miner Res.

[B28] Zhou Y, Dai DL, Martinka M, Su M, Zhang Y, Campos EI, Dorocicz I, Tang L, Huntsman D, Nelson C, Ho V, Li G (2005). Osteopontin expression correlates with melanoma invasion. J Invest Dermatol.

[B29] Rangel J, Nosrati M, Torabian S, Shaikh L, Leong SP, Haqq C, Miller JR, Sagebiel RW, Kashani-Sabet M (2008). Osteopontin as a molecular prognostic marker for melanoma. Cancer.

[B30] Breslow A (1970). Thickness, cross-sectional areas and depth of invasion in the prognosis of cutaneous melanoma. Ann Surg.

[B31] Clark WH, From L, Bernardino EA, Mihm MC (1969). The histogenesis and biologic behavior of primary human malignant melanomas of the skin. Cancer Res.

[B32] Straume O, Akslen LA (2001). Expresson of vascular endothelial growth factor, its receptors (FLT-1, KDR) and TSP-1 related to microvessel density and patient outcome in vertical growth phase melanomas. Am J Pathol.

[B33] Kononen J, Bubendorf L, Kallioniemi A, Barlund M, Schraml P, Leighton S, Torhorst J, Mihatsch MJ, Sauter G, Kallioniemi OP (1998). Tissue microarrays for high-throughput molecular profiling of tumor specimens. Nat Med.

[B34] Straume O, Akslen LA (2002). Importance of vascular phenotype by basic fibroblast growth factor, and influence of the angiogenic factors basic fibroblast growth factor/fibroblast growth factor receptor-1 and ephrin-A1/EphA2 on melanoma progression. Am J Pathol.

[B35] Nocito A, Bubendorf L, Maria Tinner E, Suess K, Wagner U, Forster T, Kononen J, Fijan A, Bruderer J, Schmid U, Ackermann D, Maurer R, Alund G, Knonagel H, Rist M, Anabitarte M, Hering F, Hardmeier T, Schoenenberger AJ, Flury R, Jager P, Luc Fehr J, Schraml P, Moch H, Mihatsch MJ, Gasser T, Sauter G (2001). Microarrays of bladder cancer tissue are highly representative of proliferation index and histological grade. J Pathol.

[B36] Straume O, Salvesen HB, Akslen LA (1999). Angiogenesis is prognostically important in vertical growth phase melanomas. Int J Oncol.

[B37] Haugland HK, Vukovic V, Pintilie M, Fyles AW, Milosevic M, Hill RP, Hedley DW (2002). Expression of hypoxia-inducible factor-1alpha in cervical carcinomas: correlation with tumor oxygenation. Int J Radiat Oncol Biol Phys.

[B38] Straume O, Akslen LA (1997). Alterations and prognostic significance of p16 and p53 protein expression in subgroups of cutaneous melanoma. Int J Cancer.

[B39] Vaupel P, Mayer A (2007). Hypoxia in cancer: significance and impact on clinical outcome. Cancer Metastasis Rev.

[B40] Rofstad EK, Mathiesen B, Henriksen K, Kindem K, Galappathi K (2005). The tumor bed effect: increased metastatic dissemination from hypoxia-induced up-regulation of metastasis-promoting gene products. Cancer Res.

[B41] Hsu MY, Shih DT, Meier FE, van Belle P, Hsu JY, Elder DE, Buck CA, Herlyn M (1998). Adenoviral gene transfer of beta3 integrin subunit induces conversion from radial to vertical growth phase in primary human melanoma. Am J Pathol.

[B42] van Belle PA, Elenitsas R, Satyamoorthy K, Wolfe JT, Guerry Dt, Schuchter L, van Belle TJ, Albelda S, Tahin P, Herlyn M, Elder DE (1999). Progression-related expression of beta3 integrin in melanomas and nevi. Hum Pathol.

[B43] Koistinen P, Ahonen M, Kahari VM, Heino J (2004). alphaV integrin promotes in vitro and in vivo survival of cells in metastatic melanoma. Int J Cancer.

[B44] Nikkola J, Vihinen P, Vlaykova T, Hahka-Kemppinen M, Heino J, Pyrhonen S (2004). Integrin chains beta1 and alphav as prognostic factors in human metastatic melanoma. Melanoma Res.

[B45] Castel S, Pagan R, Garcia R, Casaroli-Marano RP, Reina M, Mitjans F, Piulats J, Vilaro S (2000). Alpha v integrin antagonists induce the disassembly of focal contacts in melanoma cells. Eur J Cell Biol.

[B46] Mitjans F, Meyer T, Fittschen C, Goodman S, Jonczyk A, Marshall JF, Reyes G, Piulats J (2000). In vivo therapy of malignant melanoma by means of antagonists of alphav integrins. Int J Cancer.

[B47] Baldi A, Santini D, Russo P, Catricala C, Amantea A, Picardo M, Tatangelo F, Botti G, Dragonetti E, Murace R, Tonini G, Natali PG, Baldi F, Paggi MG (2004). Analysis of APAF-1 expression in human cutaneous melanoma progression. Exp Dermatol.

[B48] Mooney EE, Ruis Peris JM, O'Neill A, Sweeney EC (1995). Apoptotic and mitotic indices in malignant melanoma and basal cell carcinoma. J Clin Pathol.

[B49] Shukuwa T, Katayama I, Koji T (2002). Fas-mediated apoptosis of melanoma cells and infiltrating lymphocytes in human malignant melanomas. Mod Pathol.

[B50] Reed JC (2003). Apoptosis-targeted therapies for cancer. Cancer Cell.

